# Hypophosphite of Soda and Quinine in Enlargement of the Spleen

**Published:** 1874-01

**Authors:** R. S. Hallsey

**Affiliations:** Williamston, North Carolina


HYPOPHOSPHITE OF SODA AND QUININE IN ENLARGEMENT OF THE SPLEEN.—In one number of your journal, some months since, I saw hypophosphite of soda and quinine recommended for chronic enlargement of the spleen. I had been using the prescription, hypophosphite of soda ten grains, and quinine one grain, three times a day for eight months before I saw the publication alluded to, but had not sufficiently tested it; but having continued to use it to the present time, I can positively assert that I have never had it to fail of effecting a perfect cure in a solitary instance, when it had been duly persevered with. Nor have I ever known it to produce the least sensible effect upon the disease, until it had been taken seventeen days; and immediately after the seventeenth day, in eleven cases, the enlargement has commenced to disappear, and in a few days the cure was complete.

R. S. HALLSEY, M.D.

WILLIAMSTON, NORTH CAROLINA.

